# Androgens Modulate Bcl-2 Agonist of Cell Death (BAD) Expression and Function in Breast Cancer Cells

**DOI:** 10.3390/ijms241713464

**Published:** 2023-08-30

**Authors:** Catia Morelli, Chiara Chiodo, Marta Claudia Nocito, Alessandro Cormace, Stefania Catalano, Diego Sisci, Rosa Sirianni, Ivan Casaburi, Sebastiano Andò, Marilena Lanzino

**Affiliations:** 1 Department of Pharmacy and Health and Nutritional Sciences, University of Calabria, 87036 Arcavacata di Rende, CS, Italy; catia.morelli@unical.it (C.M.); martaclaudia.nocito@unical.it (M.C.N.); stefania.catalano@unical.it (S.C.); diego.sisci@unical.it (D.S.); rosa.sirianni@unical.it (R.S.); sebastiano.ando@unical.it (S.A.); 2 Centro Sanitario, University of Calabria, Via P. Bucci, 87036 Arcavacata Di Rende, CS, Italy; chiara.chiodo@unical.it (C.C.); alessandro.cormace@unical.it (A.C.)

**Keywords:** breast cancer, androgens, AR, BAD, MCF-7, mitochondria, cyclin D1

## Abstract

Androgen receptor (AR) expression in estrogen receptor-positive (ER+) breast cancer (BC) correlates with lower tumor grade and a better clinical outcome. Additionally, in normal mammary epithelium or ER+ BC preclinical models, androgens counteract basal/ER-dependent proliferation. Here, we report an additional mechanism, underlining the protective role exerted by AR. Specifically, the activation of intracellular AR upregulates the Bcl-2-family protein BAD, and TCGA database analyses show that in ER+ BC, BAD expression is associated with better disease-free survival. Ligand-activated AR influences its own and BAD cellular compartmentalization by enhancing levels in the nucleus, as well as in mitochondrial fractions. In both compartments, BAD exerts unconventional functions. In the nucleus, BAD and AR physically interact and, upon androgen stimulation, are recruited at the AP-1 and ARE sites within the cyclin D1 promoter region, contributing to explaining the anti-proliferative effect of androgens in BC cells. Androgens cause an enrichment in BAD and AR content in the mitochondria, correlated with a decrease in mitochondrial function. Thus, we have defined a novel mechanism by which androgens modulate BAD expression, its mitochondria localization, and nuclear content to force its ability to act as a cell cycle inhibitor, strengthening the protective role of androgen signaling in estrogen-responsive BCs.

## 1. Introduction

Although breast cancer is a heterogeneous disease [1] with different behavior, outcome, and response to therapy, breast tumorigenesis is considered a hormone-driven transformation process [2,3]. As a consequence, the expression status of estrogen receptor (ER), progesterone receptor (PR), and human epidermal growth factor receptor 2 (HER2) define prognostic differences in patient outcomes, resulting in routine clinical stratification of tumors [4,5]. Although the presence of AR in normal and neoplastic breast tissues was revealed decades ago [6,7,8], only in recent years the role played by androgens/androgen receptor (AR) signaling in breast cancer growth and progression, prognosis, and treatment has gained much interest. Consequently, AR signaling has been analyzed, mainly for the definition of new tailored therapies [9,10,11].

AR expression/action in breast tumors appears to be clinically relevant and disease-context-specific [12]. AR is the most widely expressed nuclear steroid receptor in all stages of breast cancer, being present in up to 90% of primary mammary tumors and 75% of metastatic tumors [13,14,15,16,17]. The frequency of AR expression differs between breast cancer subtypes, varying from 85–95% in ER-positive (ER+) to 15–75% in ER-negative (ER−) breast tumors [18,19,20,21,22,23,24,25,26]. Moreover, in ER+ primary breast cancers, AR positivity correlates with lower tumor grade, burden, and Ki67 labeling index, as well as with a better clinical outcome [27,28,29,30]. Indeed, AR expression is associated with a better outcome in ER+ breast cancer patients, including high-risk ER+ cancers undergoing endocrine therapy. The latter observation emphasizes a putative role for AR as a positive predictive biomarker of response to endocrine therapy in these subsets of breast tumors [31]. Androgens’ ability to counteract estrogens/ER-dependent tumor growth has been observed in both normal mammary epithelium [32] and preclinical models of ER+ breast cancer [31,33,34,35,36,37,38,39,40]. In both contexts, the pro-apoptotic effects exerted by androgen/AR-dependent have been extensively evidenced [35,36,41,42].

The dysregulation of programmed cell death is critical for cancer cell survival and cancer development. There are numerous mechanisms adopted by cancer cells to override barricades that otherwise would cause apoptosis. These mechanisms include blockage of apoptosis by activation of oncogenes such as Myc and Cyclin D1 [43], as well as amplification of the anti-apoptotic apparatus, downregulation of the pro-apoptotic program, or both [44]. The members of the Bcl-2 family of proteins, which includes proteins acting as blockers and others working as promoters of cell death, are fundamental regulators of apoptosis [43].

Interestingly, several Bcl-2 family members exert regulatory functions that go beyond the modulation of apoptosis [45,46]. Some, by acting as metabolic regulators, are implicated in metabolic reprogramming in cancer development [47,48,49]; some others may influence cancer cell cycle progression [50], as well as several crucial factors governing the epithelial–mesenchymal transition [51].

In particular, the BH3-only pro-apoptotic member BAD connects the cell-survival signaling pathway to apoptosis [52,53]. BAD represents a central element in many types of cancer, including breast carcinomas [54,55,56,57,58,59], and is a strong predictor of overall survival [60,61,62]. BAD shows a highly tissue-specific distribution and, interestingly, its expression is higher in normal human breast compared to other organs [63]. Remarkably, even though only limited data are available, in ER+ human breast cancers, higher BAD expression is associated with better disease-free survival [58]. In contrast, a lack of BAD expression correlates with lymph node metastasis and higher tumor grade [60]. These observations suggest an important and protective role for BAD in primary breast tumors. However, the mechanism(s) linking BAD to the pathobiology of human breast cancer is (are) yet to be resolved.

Hence, in this study, we aimed to further clarify and analyze the specific signal transduction pathway(s) by which androgens/AR exert their protective, anti-proliferative/pro-apoptotic effect in ER+ breast tumors. In this context, we demonstrated the existence of a novel, additional mechanism through which androgen administration maintains high BAD levels and, more interestingly, triggers a number of unconventional BAD functions. Our reports contribute to better explain the inhibitory role of androgen in breast tumor cell proliferation.

## 2. Results

### 2.1. Androgens Increase BAD Expression, Which Affects Breast Cancer Patients’ Survival

The unaromatizable androgen Dihydrotestosterone (DHT) and the synthetic agonist of AR Mibolerone (Mb) inhibit cell proliferation and trigger apoptosis in ER+ breast cancer cells by activating AR [35,41].

**Figure 1:**
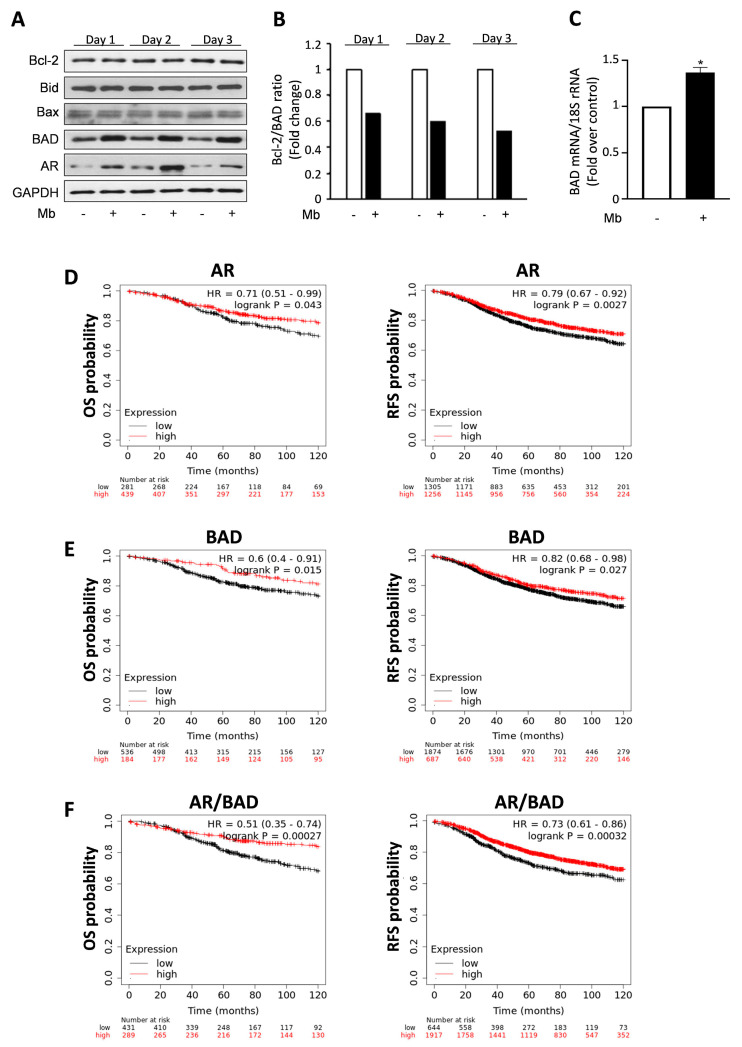
BAD and AR relative expression in MCF-7 cells and breast cancer patients. (**A**) Western blotting analysis of protein lysates from MCF-7 breast cancer cells treated with vehicle (-) or 10 nM Mb for 1, 2, and 3 days. (**B**) Band intensities from panel A were measured and normalized to the relative GAPDH content. Histogram represents the normalized Bcl-2/BAD ratio. (**C**) Quantitative Real-Time RT-PCR from MCF-7 cells treated with vehicle (-) or 10 nM Mb for 24 h. BAD mRNA expression was normalized to 18S rRNA content. Data represent the mean ± S.D. of three separate experiments, each performed in triplicate. * *p* < 0.05 vs. vehicle. (**D**–**F**) Overall survival (OS) and relapse-free survival (RFS) were evaluated in a cohort of ER+ BC patients. Kaplan–Meier analysis was performed regardless of specific treatments. Kaplan–Meier was plotted for high (above median, in red) and low (below median, in black) AR (**D**), BAD (**E**) and concomitant AR/BAD (**F**) expression. Biased and outlier data were excluded from the analysis. Hazard-ratios were calculated at the best auto-selected cut-off, and *p*-values were calculated using the log rank test.

Thus, we aimed to better clarify the underlying mechanisms by which androgen/AR signaling exerts anti-proliferative/pro-apoptotic effects in breast cancer cells. Then, the consequence of Mb treatment on the expression of several pro- and anti-apoptotic proteins was investigated in ER+ MCF-7 breast cancer cells. As shown in Figure 1A, androgen administration induced a significant increase in the expression of the pro-apoptotic protein BAD that was evident after 24 h exposure and persisted thereafter. As a consequence, the Bcl-2/BAD ratio was reduced, altering the delicate balance between cell death inhibitors and inducers (Figure 1B). Upregulated BAD expression by Mb treatment was also observed at mRNA levels, as evidenced using qReal-Time PCR (Figure 1C).

To better elucidate the role of AR and BAD cooperation, we investigated if our findings may have an impact on the outcome of ER+ breast cancer patients [64]. Low expression levels of AR and BAD are associated with decreased overall survival (OS) and relapse-free survival (RFS) in ER+ breast cancer, while their high levels appear to be protective, as Kaplan–Meier survival analyses show (Figure 1D,E). Moreover, ER+ patients’ database was analyzed for concomitant AR and BAD expression, evidencing that high co-expression of both genes is a prognostic factor of better outcomes in terms of both OS and RFS (Figure 1F). These findings further support the use of AR agonists as adjuvant therapy for ER+ breast cancer patients.

### 2.2. Androgens Influence BAD Cellular Compartmentalization in Breast Cancer Cells

**Figure 2:**
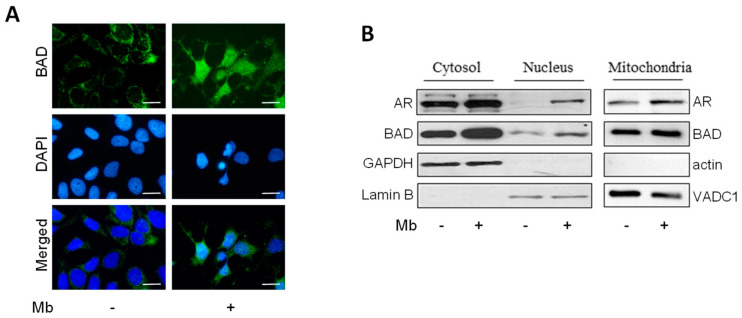
Androgens induce BAD nuclear translocation in breast cancer cells. (**A**) Immunofluorescence assay for BAD (green) was performed in MCF-7 cells treated with 10 nM Mb for 24 h. DAPI staining was used for nuclei detection. Bar = 20 μm (**B**) Western blotting analysis of cytoplasmic, nuclear, and mitochondrial protein fractions from MCF-7 breast cancer cells treated for 24 h with vehicle (-) or 10 nM Mb as indicated. Lamin B, GAPDH, actin, and VADC1 were used as a loading control.

Immunofluorescence analysis was performed in MCF-7 cells detecting, in the cytoplasm and perinuclear region, clear BAD immunoreactivity, which was enhanced following Mb administration. Yet more interesting was the observation that the fluorescent signal appeared to be markedly increased in the nuclear compartment, suggesting that androgen treatment may cause the nuclear localization of BAD, as further evidenced in images from merged DAPI and BAD signals (Figure 2A). To confirm BAD translocation into the nuclear compartment of MCF7 cells following Mb administration, cell fractionation to separate nuclear and cytoplasmic proteins was performed. As demonstrated by Western blotting analysis, in untreated cells, BAD was mainly present in the cytoplasmic fraction, while following Mb treatment, its nuclear amount significantly increased. As expected, Mb treatment increased AR protein levels, as well as its nuclear translocation (Figure 2B). Similar patterns of BAD cellular levels and localization were also observed in ER+/AR+ T47D and ZR75, as well as in ER−/AR+ SKBR3 breast cancer cells (Figure S1A–C). These results strongly suggest a nuclear role for BAD upon androgen stimulation. Additionally, mitochondrial fractions were also analyzed for BAD and AR abundance, and it was observed that they were enriched in both AR and BAD content (Figure 2B).

### 2.3. AR Activation Affects Mitochondria Functions

**Figure 3:**
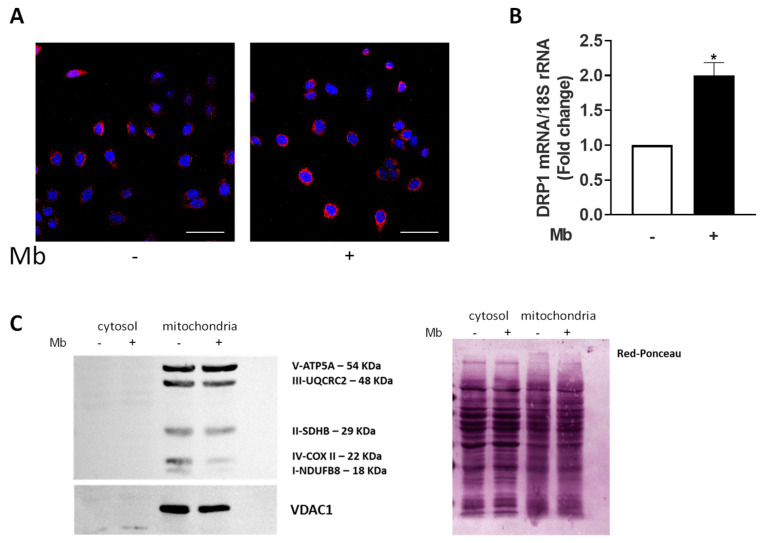
Ligand-activated AR impairs mitochondria functions. (**A**) MCF-7 cells treated with vehicle (-) or 10 nM Mb were stained with 200 nM MitoTracker Red CMXRos (CMXRos). DAPI was used to stain nuclei. The fluorescent signal was analyzed using an FV3000 confocal laser scanning microscope (Olympus Corporation, Tokyo, Japan). Bar = 50 μm (**B**) Quantitative RNA from MCF-7 cells treated with vehicle (-) or 10 nM Mb for 24 h was analyzed using Real-Time RT-PCR for DRP1 and normalized to 18S rRNA content. Data represent the mean ± S.D. of three separate experiments, each performed in triplicate. * *p* ≤ 0.01. (**C**) Western blotting analysis of OXPHOS and VDAC1 in mitochondrial protein fractions from MCF-7 breast cancer cells treated for 24 h with vehicle (-) or 10 nM Mb. Cytoplasmic proteins demonstrate no signal. Red Ponceau demonstrates equal loading.

A deeper analysis showed that activated AR affected the bioenergetic cell behavior. To evaluate the mitochondrial network morphology, MCF-7 cells were stained with the mitochondria-targeting dye MitoTracker Red CMXRos and analyzed under a fluorescence microscope. As depicted in Figure 3A, Mb administration caused an enhancement of ROS signal that, presumably, could be attributed to increased oxidative stress, coupled with an effort to increase mitochondrial fission. Indeed, as indicated by qReal-Time PCR, Mb administration also increased Drp1 mRNA levels (Figure 3B), a key player in mitochondrial network dynamics [65]. Moreover, upon Mb treatment, a concomitant decrease in OXPHOS content was also observed (Figure 3C).

Together, these data indicate an attempt initiated by breast cancer cells, in response to androgens, to control the number of functional mitochondria.

### 2.4. BSA-Conjugated Testosterone Induces Apoptosis without Influencing BAD Nuclear Translocation

Several observations indicate that, in addition to the classical intracellular AR, membrane androgen receptors (mAR) are also involved in the regulation of cell growth, motility, and death by apoptosis in a number of tumors, including breast cancer. These effects may occur independently of the intracellular AR [66,67,68,69,70].

**Figure 4:**
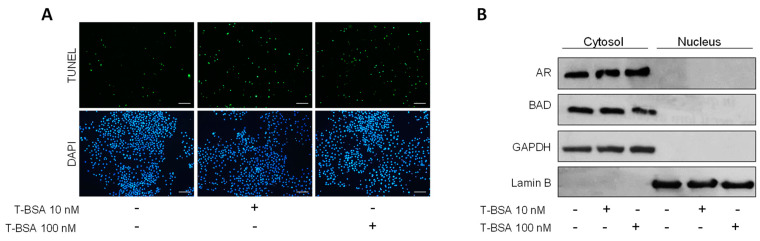
BSA-conjugated testosterone induces apoptosis without influencing BAD cellular compartmentalization. (**A**) MCF-7 cells treated with 10 nM or 100 nM T-BSA for 6 days were subjected to TUNEL nuclear staining and observed under a fluorescence microscope. DAPI staining was used for nuclei detection. Bar = 100 µm (**B**) Cytosol/nuclear protein fractions from MCF-7 cells treated with vehicle (-), and T-BSA (10, 100 nM) for 24 h. Lamin B and GAPDH were used as loading control.

Therefore, the effect exerted on MCF-7 breast cancer cells by treatment with testosterone-BSA (T-BSA), a testosterone analog that does not penetrate the cell membrane, was also evaluated. Unsurprisingly, treatment with T-BSA increased apoptotic nuclei compared to control, as indicated by TUNEL assay (Figure 4A). Nevertheless, T-BSA administration was not able to induce nuclear translocation of either BAD or AR (Figure 4B). These data strongly suggest that, following androgen treatment, BAD cellular localization and function relies on the activation of the classical intracellular AR.

### 2.5. Mibolerone Induces the Formation of an AR/BAD Complex and Influences BAD Recruitment at the AP-1 and ARE Sites on the Cyclin D1 Gene Promoter

**Figure 5:**
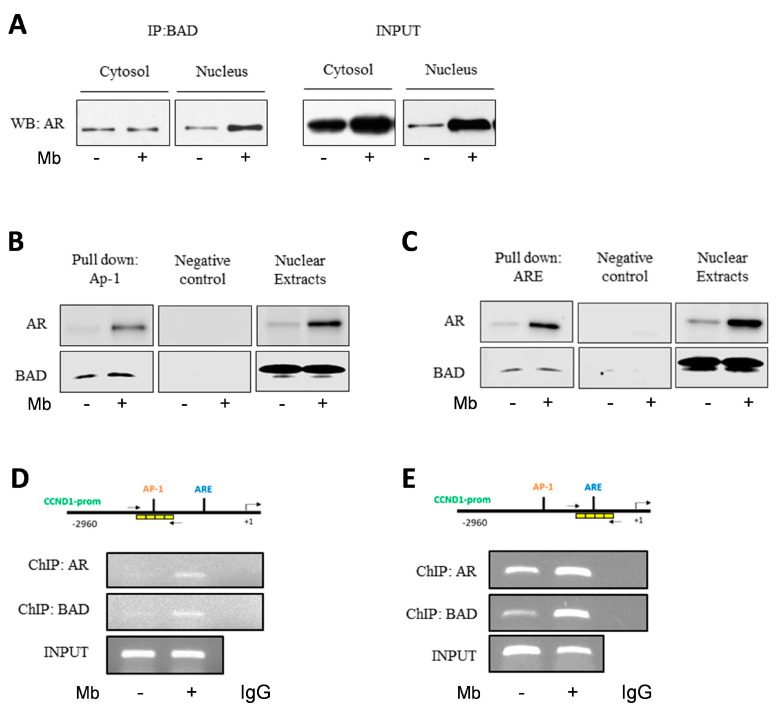
Mibolerone induces the formation of an AR/BAD complex and influences BAD recruitment at the AP-1 and ARE sites on the cyclin D1 gene promoter. (**A**) Cytosol and nuclear lysates from MCF-7 cells treated with 10 nM Mb for 24 h were immunoprecipitated with anti-BAD antibody and immunoblotted to detect AR protein levels. (**B**–**C**) Nuclear extract from MCF7 cells treated with 10 nM Mb or vehicle (-) for 2 h were incubated with biotinylated oligonucleotides containing the *CCND1*-AP-1 and -ARE sites and subjected to DAPA. Bound proteins were subjected to Western blotting analysis using anti-AR or anti-BAD antibodies. The unbound fraction was loaded as negative control; nuclear extracts were loaded as positive control. (**D**–**E**) Chromatin from MCF-7 cells treated with 10 nM Mb or vehicle (-) for 2 h was precipitated using anti-AR or anti-BAD antibodies. PCR was carried out using primers amplifying the region indicated by arrows and analyzed by agarose gel electrophoresis. IgG indicates negative control samples. DNA input indicates the loading control.

To assess whether androgen-regulated BAD nuclear localization could require direct interaction with AR, a co-immunoprecipitation assay was performed using nuclear and cytoplasmic proteins extracted from vehicle- and Mb-treated MCF-7 cells. A constitutive association between AR and BAD was present in the cytoplasm as well as in the nuclear fraction of vehicle-treated cells. In contrast, AR/BAD complex abundance clearly increased in the nucleus upon Mb treatment (Figure 5A). We then investigated the biological significance of the nuclear AR/BAD complex. It has been previously demonstrated that nuclear BAD influences breast cancer cell-cycle progression by preventing cyclin D1 transcription. This event requires a negative regulation of c-Jun at an AP-1 site within the cyclin D1 gene promoter [50]. In addition, cyclin D1 is an AR target gene, downregulated by androgens in ER+ breast cancer cells [36]. Thus, we investigated whether AR and BAD could cooperate at the cyclin D1 promoter by performing DNA affinity precipitation assay (DAPA). To this aim, double-stranded oligonucleotides containing AP-1 (Figure 5B) or ARE (Figure 5C) sequences of the cyclin D1 promoter were used. BAD was bound to the AP-1 sequence in control lysates, as previously demonstrated [50], and this association increased following Mb treatment (Figure 5B). Moreover, a band indicating BAD association to the ARE consensus sequence was also present (Figure 5C). As expected, endogenous AR associated with the ARE consensus oligonucleotide in basal conditions and its abundance increased in Mb-treated samples [36] (Figure 5C). Interestingly AR association to the AP-1 consensus oligonucleotide was also present (Figure 5B). Furthermore, a similar pattern of AR/BAD binding to the ARE site from the cyclin D1 promoter was observed in T47D breast cancer cells (Figure S2). These results suggest that Mb administration induces the formation of a nuclear AR/BAD complex able to bind to the ARE and AP-1 sites of the cyclin D1 promoter. This observation was further confirmed by ChIP assay using anti-AR or anti-BAD antibodies: AR and BAD do interact with cyclin D1 promoter on the native chromatin. Indeed, both AR and BAD occupancy of either the ARE or AP-1 consensus sequences within the cyclin D1 promoter increased upon Mb administration (Figure 5D,E).

## 3. Discussion

In this study, we report a novel, additional, mechanism that contributes to explain the protective, anti-proliferative role exerted by androgens/AR signaling in ER+ breast cancers. This mechanism involves the modulation of the amount, cellular distribution, and function of BAD, a BH3-only pro-apoptotic member of the Bcl-2 family proteins.

Tumor growth and progression are linked to cancer cells’ ability to acquire several “hallmarks of cancer”, including escape from apoptosis, through deregulation of the BCL-2 family members [71,72]. Cancer cells are able to bypass apoptotic stimuli through a number of mechanisms, including the upregulation of anti-apoptotic or the loss of pro-apoptotic proteins [73]. The downregulation or inactivation of pro-apoptotic BH3-only proteins in breast cancer contributes to the development of therapy-unresponsive cancer phenotypes. On the contrary, their over-expression contributes to sensitize tumor cells to various anti-cancer drugs [58,59,74].

Our data indicate that, in ER+ breast cancer cells, the expression of the BH-3-only protein BAD is influenced by AR-dependent signaling, since its cellular levels appear to be significantly increased upon androgen administration. BAD upregulation via activated AR may assume a significant relevance, as tumor-suppressor potential has been reported for several BH3-only proteins, consistently with their pro-apoptotic role [75,76]. It is noteworthy that normal mammary tissues express higher BAD levels than breast tumors [51,59]. Additionally, the BAD encoding gene is downregulated in breast cancers from patients who developed metastasis, and its downregulation is statistically associated with positive lymph node status, advanced pathological stages, and tumor size [77]. Conversely, in primary ER+ breast cancers, higher BAD expression sensitizes cells to chemotherapy [57] and is related to a significantly better disease-free survival [58,60].

Remarkably, ligand-activated AR regulates BAD intracellular localization by inducing its translocation inside the nucleus. This was observed for ER+ cells, MCF7, T47D and ZR75, as well as for ER- SKBR3 cells. Subcellular compartmentalization is a central feature of eukaryotic cells as it permits the physical segregation and parallel accomplishment of a number of distinct biochemical processes inside the cell [78]. For instance, Bcl-2 family members have been found in the mitochondria, endoplasmic reticulum, Golgi Apparatus, peroxisomes, and nucleus [79,80,81,82,83], evidencing their pleiotropic functions within the cell.

Classically, steroid hormones bind to receptors localized in the cytosol, inducing their translocation to the nucleus. Here, they act as transcription factors by binding to specific DNA response elements [84]. It is now well accepted that, in addition to this classic mechanism of action, steroid hormones can induce a non-nuclear rapid signaling response [85,86,87,88]. This signaling is mediated by receptors mainly localized on the cell membrane [66,67,68,69,70], which, once activated, trigger signaling cascades that involve the formation of a number of secondary messengers and the activation of kinases or phospholipases [89,90]. The use of BSA-bound hormone, which does not cross the cell membrane, allows discrimination between classic and membrane-initiated hormone signals. In our experimental models, BSA-conjugated testosterone was able to trigger apoptosis, as is already well documented [66,68,69]. In contrast, T-BSA did not influence BAD cellular localization, evidencing a central role of intracellular AR in mediating BAD compartmentalization upon Mb administration.

Then, AR seems to have a dual mechanism of action: (1) it increases BAD mRNA and consequently protein, and (2) it is directly involved in BAD shuttling. The first mechanism could require AR interaction with the BAD promoter, since a preliminary sequence analysis revealed the presence of a putative ARE site (5′-TGTCCT-3) located at −19 bp from the ATG translational starting site (unpublished data). The second mechanism requires AR/BAD physical interaction and occurs when AR is activated by its ligand. This mechanism is proved by immunostaining and Western blotting experiments in cells treated with Mibolerone, manifesting a remarkable nuclear positive signal for BAD.

Within the nuclear compartment, BAD would exert “non-canonical” roles. A role for BAD, not only restricted to cell death promotion, is actually an emerging concept [49,91,92]. In ER+ breast cancer cells, BAD over-expression reduces cell growth [50] and prevents cancer cell metastatic effusion by downregulating proteins that mediate epithelial-to-mesenchymal transition [51]. The reported androgen-dependent nuclear localization of BAD might further explain the protective action exerted by androgens against the growth of breast cancer cells. We previously demonstrated that ligand-activated AR is able to inhibit the transcription of cyclin D1 by binding to a specific ARE sequence present on the proximal promoter of the CCDN1 gene [36]. We now add a new piece to the puzzle to help explain the complex mechanisms through which androgens influence breast cancer cell biology. Indeed, as a consequence of AR activation, BAD is forced to perform its role as a cell cycle inhibitor. This unconventional role for BAD was demonstrated in MCF-7 cells, in which nuclear BAD negatively regulates cyclin D1 expression, reducing the G1/S transition of the cell cycle. The molecular mechanism underlying this inhibition involves BAD binding to the AP1 site within the proximal promoter of the cyclin D1 gene. Through this mechanism of action, nuclear BAD interferes with cyclin D1 transcription induced by estrogens and/or growth factors [50]. Our experiments deepen the knowledge of this mechanism, since we observed that Mibolerone administration induces the formation of an AR/BAD nuclear complex and their recruitment on the AP1 and ARE sites within the cyclin D1 promoter, as proven using both DAPA and ChIP assays.

Interestingly, in control cells, BAD has a punctuate cytoplasmic distribution, consistent with organelle-specific localization. It has been reported that in the mitochondria, BAD exerts unconventional roles by stimulating complex I activity, favoring tumor growth [49]. Remarkably, Mibolerone caused an increase in AR and BAD mitochondrial content; however, the observation that androgen treatment decreases OXPHOS abundance implicates a limitation of BAD’s effects on COXI. Our previous reports demonstrated that Mibolerone causes a reduction in breast cancer cell viability [36], as evaluated using MTT assay, a method based on the reduction of MTT into purple formazan granules within healthy and active mitochondria. Then, the decrease in OXPHOS levels following androgens exposure could mechanistically explain our previous observation on cell viability and implicate a possible repressive effect of AR on mitochondrial DNA transcription. Another relevant observation is the upregulation of DRP-1 mRNA following Mb administration. DRP-1 regulates mitochondrial fission, an event whose role during cell death has been long debated. Mitochondrial fragmentation could facilitate cytochrome c release and apoptosis or, alternatively, could represent a survival attempt to overcome cell death [93]. Androgen’s ability to increase DRP-1 amount has been observed in prostate cancer [94], where, however, in contrast to breast cancer, AR has a negative prognostic value. Thus, the role of AR-initiated fission in breast cancer deserves further studies.

The clinical relevance of our data is supported by Kaplan–Meier survival analyses. KM plots indicate that higher levels of AR and BAD have, individually, a positive impact on the OS and RFS of ER+ breast cancer patients. More importantly, significantly longer OS and RFS were seen in patients expressing high AR when co-expressed with BAD. Thus, these observations support the hypothesis that the inclusion of androgen-like molecules in the therapeutic setting could increase BAD and AR expression and improve ER+ breast cancer patients’ outcomes.

With this study, we add an additional piece of information about the complex mechanisms exerted by androgens on breast cancer. As a consequence of AR activation, BAD is forced to perform its role as a cell cycle inhibitor by negatively modulating cyclin D1 gene transcription. Additionally, mitochondrial AR localization, by reducing OXPHOS levels, may prevent the potential stimulatory effects exerted by BAD on complex I activity, shown to promote tumor growth.

## 4. Materials and Methods

### 4.1. Reagents and Antibodies

Mibolerone (Mb) was from Sigma Aldrich (St. Louis, MO, USA). The antibodies against β-Actin (AC-15), BAD (C-7), BAX (B-9), Bcl-2 (C-2), BID (FL-195), GAPDH (FL-335), and Lamin B (C-20) were from Santa Cruz Biotechnology (Bolivia); OXPHOS and VDAC1 were from Abcam (Cambridge, UK); and AR (D6F11) was from Cell Signaling (Boston, MA, USA).

### 4.2. Cell Cultures

Human breast cancer MCF-7, T47D, and SKBR3 were purchased from American Type Culture Collection (ATCC). Growth-medium components for each cell line are reported in Table 1. All cell lines were regularly tested for mycoplasma negativity (MycoAlert Mycoplasma Detection Assay). For treatment, Mibolerone was added in phenol-red-free (PRF) medium containing 2.5% charcoal-stripped serum.

### 4.3. Western Blot Analysis

Total cell proteins and the cytoplasmic and nuclear fractions were obtained from 70% confluent cell cultures. Western blotting (WB) was performed as previously described [95]. Blots were incubated with primary antibodies (overnight, 4 °C) and then with appropriate horseradish-peroxidase-conjugated secondary antibodies (1 h, room temperature). Immunoreactive bands were detected using the ECL Western blotting detection system (Santa Cruz Biotechnology (Dallas, TX, USA), sc-2048). Images were captured using UltraCruz Autoradiography Film (Santa Cruz Biotechnology) or iBright Imaging System (Thermo-Fisher (Waltham, MA, USA). The images from films were acquired using an Epson Perfection scanner (Epson, Japan) using Photoshop software (Adobe) (https://www.adobe.com/au/products/photoshop.html, accessed on 20 July 2023). The optical densities of the spots were analyzed by using ImageJ software (NIH) (https://imagej.en.softonic.com/download, accessed on 20 July 2023).

### 4.4. Total RNA Extraction, Reverse Transcription Polymerase PCR and Real-Time RT-PCR Assay

Total RNA was extracted from MCF-7 cells using the TRIzol reagent, and cDNA was synthesized using a reverse transcription polymerase chain reaction (PCR) method using a RETROscript kit. The expression of selected genes was quantified using Real-Time PCR, as previously described [36], using the primers (Invitrogen) (Waltham, MA, USA) reported in Table 2. Assays were performed in triplicate.

### 4.5. Immunoprecipitation

Total proteins and the cytoplasmic and nuclear fractions were obtained from 70% confluent cells. Immunoprecipitation was performed as previously described [95]. Briefly, primary Ab was incubated with protein A/G agarose (Santa Cruz Biotechnology) at 4 °C for 2 h in PBS buffer. In negative control samples, the primary antibody was substituted with IgG. Then, protein lysates were added and incubated at 4 °C overnight. The immune-precipitated proteins were washed with PBS buffer and separated on 11% polyacrylamide denaturing gel as described for Western blotting.

### 4.6. Chromatin Immunoprecipitation (ChIP) Assay and PCR/Real-Time PCR ChIP

MCF-7 cells were grown in 15 cm dishes to 50–60% confluence, shifted to PRF for 24 h, and then treated with 10 nM Mb or vehicle (-) for 2 h in PRF-CT. Thereafter, ChIP assay was carried out as previously described [36]. Immuno-cleared chromatin was precipitated with anti-AR or anti-BAD antibody. Immunoprecipitated DNA was analyzed through PCR using a 2 µL volume of each sample. The following primers (Invitrogen) spanning the ARE site or the AP-1 site of the proximal Cyclin D1 promoter were used: ARE forward 5′-TACCCCTTGGGCATTTGCAACGA-3′; ARE reverse 5′ACAGACGGCCAAAGAATCTCA-3′; AP-1 forward 5′-CTGCCTTCCTACCTTGACCA-3′; and AP-1 reverse 5′-TGAAGGGACGTCTACACCCC-3′. Amplification products were analyzed on a 2% agarose gel and visualized with ethidium bromide staining. The specificity of reactions was ensured using normal mouse IgG (Santa Cruz Biotechnology).

### 4.7. TUNEL Assay

Cells (3 × 10^5^) were seeded on coverslips in 35 mm Petri dishes and treated as described for growth experiments. Apoptosis was evaluated by enzymatic labeling the DNA strand breaks using a Dead End Fluorimetric TUNEL System (Promega, Milan, Italy) as previously described [35]. DAPI was used to counterstain the nuclei. Apoptotic cells were photographed at 10× magnification using an Olympus dp50 camera and ViewFinder software (https://steamunlocked.net/6-viewfinder-free-download/, accessed on 20 July 2023).

### 4.8. DNA Affinity Precipitation Assay (DAPA)

Nuclear extracts were obtained from cells stimulated with 10 nM Mb or vehicle (-) for 2 h. DAPA was performed as previously described [36]. The DNA sequences were prepared by annealing a biotinylated sense oligonucleotide (for ARE, 5-[Bio]-GCTAAATTAGTTCTTGCAATTTAC-3; for AP-1, 5-[Bio]-AATGAGTCAGAATGGAGA-3) with nonbiotinylated antisense oligonucleotide (for ARE, 5-GTAAATTGCAAGAACTAATTTAGC; for AP-1, 5- GTGATCTCCCATTCTGACTCATT-3). Supernatants containing the unbound fraction were loaded on gel and used as negative controls.

### 4.9. Immunofluorescence Assay

Immunofluorescence assay was performed as previously described [35] with minor modifications. Briefly, cells platelet on 12 mm glass coverslips were fixed with 4% paraformaldehyde and permeabilized using 0.2% Triton X-100, followed by BSA blocking and incubated with anti-BAD antibody at 4 °C overnight and then with fluorescein-conjugated secondary at room temperature for 1 h. DAPI (Sigma) staining was used for nuclei detection. Fluorescence was evaluated using an Olympus BX51 fluorescence microscope at 100× magnification, and cells were photographed using ViewFinder software (https://steamunlocked.net/6-viewfinder-free-download/, accessed on 20 July 2023) with an Olympus camera system dp50.

### 4.10. Mitotracker Red CMXRos Staining

MitoTracker™ Red CMXRos was prepared as per the manufacturer’s recommendation (Invitrogen, USA). CMXRos dye was dissolved in DMSO at a concentration of 1 mM and stored at –20 °C until use. A 200 nM working solution in growth medium was prepared prior to staining and added to cultured cells for 45 min. After treatment, cells were washed with PBS two times and fixed with 4% PFA for 10 min at room temperature. After that, cells were washed with PBS and incubated with 0.2% Triton solution for 3 min. Cells were washed again and incubated with DAPI (0.2 mg/mL) for 5 min. Finally, cells were washed again, and coverslips were mounted onto glass slides. The fluorescent signal was analyzed using an FV3000 confocal laser scanning microscope (Olympus Corporation, Tokyo, Japan).

### 4.11. Kaplan–Meier Analysis

The prognostic value of AR/BAD in breast cancer was evaluated by performing a Kaplan–Meier (K-M) analysis using the most updated version of a publicly available microarray database from breast cancer patients (https://kmplot.com/analysis/index.php?p=service&cancer=breast#, accessed on 20 July 2023). Relapse-free survival (RFS) and overall survival (OS) were evaluated in a cohort of ER+ patients, regardless of therapeutic interventions. The *p*-values were calculated using the log rank test.

### 4.12. Statistical Analysis

Statistical analysis was performed using ANOVA followed by Newman–Keuls testing to determine differences in means. All data are reported as the mean ± SD of three different experiments, each performed in triplicates; * *p* ≤ 0.05 vs. control.

## 5. Conclusions

Our results clearly show that in breast cancer cells, ligand-activated intracellular AR upregulates BAD expression and causes its translocation in the nuclei. Consequently, both proteins are engaged to the promoter region of the cyclin D1 gene. These results highlight an unconventional, apoptosis-unrelated, BAD function that can be included with other additional non-canonical roles played by this protein in different cell compartments, such as the mitochondria. BAD abundance in the mitochondria is increased in response to androgens, which also induce AR translocation in this organelle. Mitochondria increase the abundance of reactive oxygen species while decreasing the amount of OXPHOS. The observation that the fission gene DRP1 increases in response to androgens while VDCA1 protein decreases suggests the possibility that cells are attempting a survival response that deserves to be further investigated.

Together, our data deepen the knowledge of AR actions in breast cancer and further support the possibility of improving the therapeutic options against ER+ breast cancers through the use of androgen-like drugs.

## Figures and Tables

**Table 1:** Cell cultured media.

Cell Line	Media	Supplements
MCF-7	DMEM (1X)	5% FBS, 1% Pen/Strep
T47D	RPMI 1640	10% FBS, 1% Pen/Strep, 0.2 mM L-Glutamine
SKBR3	RPMI 1640 phenol red-free	10% FBS, 1% Pen/Strep, 0.2 mM L-Glutamine, 0.2 Units/mL insulin

**Table 2:** Real-Time PCR primer sequences.

Gene	Forward	Reverse
BAD	5′-GGAGGATGAGTGACGAGTTTGTG-3′	5′-GGGTGGAGTTTCGGGATGT-3′
DRP1	5′-GAGAGGTAGATCCAGATGGT-3′	5′-CCCTTCCCATCAATACATCC-3′
18S	5′-CGGCGACGACCCATTCGAAC-3′	5′-GAATCGAACCCTGATTCCCCGTC-3′

## Data Availability

Not applicable.

## Supplementary Materials

The following supporting information can be downloaded at https://www.mdpi.com/article/10.3390/ijms241713464/s1.

## References

[1] Perou C.M., Sørlie T., Eisen M.B., van de Rijn M., Jeffrey S.S., Rees C.A., Pollack J.R., Ross D.T., Johnsen H., Akslen L.A. (2000). Molecular portraits of human breast tumours. Nature.

[2] Henderson B.E., Feigelson H.S. (2000). Hormonal carcinogenesis. Carcinogenesis.

[3] Yager J.D., Davidson N.E. (2006). Estrogen carcinogenesis in breast cancer. N. Engl. J. Med..

[4] Anderson K.N., Schwab R.B., Martinez M.E. (2014). Reproductive risk factors and breast cancer subtypes: A review of the literature. Breast Cancer Res. Treat..

[5] Hammond M.E., Hayes D.F., Dowsett M., Allred D.C., Hagerty K.L., BADve S., Fitzgibbons P.L., Francis G., Goldstein N.S., Hayes M. (2010). American Society of Clinical Oncology/College of American Pathologists guideline recommendations for immunohistochemical testing of estrogen and progesterone receptors in breast cancer. J. Clin. Oncol. Off. J. Am. Soc. Clin. Oncol..

[6] Wagner R.K., Gorlich L., Jungblut P.W. (1973). Dihydrotestosterone receptor in human mammary cancer. Acta Endocrinol. Suppl..

[7] Horwitz K.B., Zava D.T., Thilagar A.K., Jensen E.M., McGuire W.L. (1978). Steroid receptor analyses of nine human breast cancer cell lines. Cancer Res..

[8] Allegra J.C., Lippman M.E., Thompson E.B., Simon R., Barlock A., Green L., Huff K.K., Do H.M., Aitken S.C. (1979). Distribution, frequency, and quantitative analysis of estrogen, progesterone, androgen, and glucocorticoid receptors in human breast cancer. Cancer Res..

[9] Chiodo C., Morelli C., Cavaliere F., Sisci D., Lanzino M. (2021). The Other Side of the Coin: May Androgens Have a Role in Breast Cancer Risk?. Int. J. Mol. Sci..

[10] You C.P., Leung M.H., Tsang W.C., Khoo U.S., Tsoi H. (2022). Androgen Receptor as an Emerging Feasible Biomarker for Breast Cancer. Biomolecules.

[11] Caswell-Jin J.L., Curtis C. (2021). Androgen receptor agonists as breast cancer therapeutics. Nat. Med..

[12] Lim E., Tarulli G., Portman N., Hickey T.E., Tilley W.D., Palmieri C. (2016). Pushing estrogen receptor around in breast cancer. Endocr.-Relat. Cancer.

[13] Lea O.A., Kvinnsland S., Thorsen T. (1989). Improved measurement of androgen receptors in human breast cancer. Cancer Res..

[14] Moinfar F., Okcu M., Tsybrovskyy O., Regitnig P., Lax S.F., Weybora W., Ratschek M., Tavassoli F.A., Denk H. (2003). Androgen receptors frequently are expressed in breast carcinomas: Potential relevance to new therapeutic strategies. Cancer.

[15] Cimino-Mathews A., Hicks J.L., Illei P.B., Halushka M.K., Fetting J.H., De Marzo A.M., Park B.H., Argani P. (2012). Androgen receptor expression is usually maintained in initial surgically resected breast cancer metastases but is often lost in end-stage metastases found at autopsy. Hum. Pathol..

[16] Honma N., Horii R., Iwase T., Saji S., Younes M., Ito Y., Akiyama F. (2012). Clinical importance of androgen receptor in breast cancer patients treated with adjuvant tamoxifen monotherapy. Breast Cancer.

[17] Ren Q., Zhang L., Ruoff R., Ha S., Wang J., Jain S., Reuter V., Gerald W., Giri D.D., Melamed J. (2013). Expression of androgen receptor and its phosphorylated forms in breast cancer progression. Cancer.

[18] Gonzalez-Angulo A.M., Stemke-Hale K., Palla S.L., Carey M., Agarwal R., Meric-Berstam F., Traina T.A., Hudis C., Hortobagyi G.N., Gerald W.L. (2009). Androgen receptor levels and association with PIK3CA mutations and prognosis in breast cancer. Clin. Cancer Res. Off. J. Am. Assoc. Cancer Res..

[19] Peters A.A., Buchanan G., Ricciardelli C., Bianco-Miotto T., Centenera M.M., Harris J.M., Jindal S., Segara D., Jia L., Moore N.L. (2009). Androgen receptor inhibits estrogen receptor-alpha activity and is prognostic in breast cancer. Cancer Res..

[20] Micello D., Marando A., Sahnane N., Riva C., Capella C., Sessa F. (2010). Androgen receptor is frequently expressed in HER2-positive, ER/PR-negative breast cancers. Virchows Arch. Int. J. Pathol..

[21] Niemeier L.A., Dabbs D.J., Beriwal S., Striebel J.M., Bhargava R. (2010). Androgen receptor in breast cancer: Expression in estrogen receptor-positive tumors and in estrogen receptor-negative tumors with apocrine differentiation. Mod. Pathol..

[22] Park S., Koo J., Park H.S., Kim J.H., Choi S.Y., Lee J.H., Park B.W., Lee K.S. (2010). Expression of androgen receptors in primary breast cancer. Ann. Oncol. Off. J. Eur. Soc. Med. Oncol..

[23] Collins L.C., Cole K.S., Marotti J.D., Hu R., Schnitt S.J., Tamimi R.M. (2011). Androgen receptor expression in breast cancer in relation to molecular phenotype: Results from the Nurses’ Health Study. Mod. Pathol..

[24] Hu R., Dawood S., Holmes M.D., Collins L.C., Schnitt S.J., Cole K., Marotti J.D., Hankinson S.E., Colditz G.A., Tamimi R.M. (2011). Androgen receptor expression and breast cancer survival in postmenopausal women. Clin. Cancer Res. Off. J. Am. Assoc. Cancer Res..

[25] Loibl S., Müller B.M., von Minckwitz G., Schwabe M., Roller M., Darb-Esfahani S., Ataseven B., du Bois A., Fissler-Eckhoff A., Gerber B. (2011). Androgen receptor expression in primary breast cancer and its predictive and prognostic value in patients treated with neoadjuvant chemotherapy. Breast Cancer Res. Treat..

[26] Yu Q., Niu Y., Liu N., Zhang J.Z., Liu T.J., Zhang R.J., Wang S.L., Ding X.M., Xiao X.Q. (2011). Expression of androgen receptor in breast cancer and its significance as a prognostic factor. Ann. Oncol. Off. J. Eur. Soc. Med. Oncol..

[27] Hickey T.E., Robinson J.L.L., Carroll J.S., Tilley W.D. (2012). Minireview: The androgen receptor in breast tissues: Growth inhibitor, tumor suppressor, oncogene?. Mol. Endocrinol..

[28] Tsang J.Y.S., Ni Y.-B., Chan S.-K., Shao M.-M., Law B.K.B., Tan P.H., Tse G.M. (2014). Androgen receptor expression shows distinctive significance in ER positive and negative breast cancers. Ann. Surg. Oncol..

[29] Ricciardelli C., Bianco-Miotto T., Jindal S., Butler L.M., Leung S., McNeil C.M., O’Toole S.A., Ebrahimie E., Millar E.K.A., Sakko A.J. (2018). The Magnitude of Androgen Receptor Positivity in Breast Cancer Is Critical for Reliable Prediction of Disease Outcome. Clin. Cancer Res..

[30] Jiang H.-S., Kuang X.-Y., Sun W.-L., Xu Y., Zheng Y.-Z., Liu Y.-R., Lang G.-T., Qiao F., Hu X., Shao Z.-M. (2016). Androgen receptor expression predicts different clinical outcomes for breast cancer patients stratified by hormone receptor status. Oncotarget.

[31] Hickey T.E., Selth L.A., Chia K.M., Laven-Law G., Milioli H.H., Roden D., Jindal S., Hui M., Finlay-Schultz J., Ebrahimie E. (2021). The androgen receptor is a tumor suppressor in estrogen receptor-positive breast cancer. Nat. Med..

[32] Peters A.A., Ingman W.V., Tilley W.D., Butler L.M. (2011). Differential effects of exogenous androgen and an androgen receptor antagonist in the peri- and postpubertal murine mammary gland. Endocrinology.

[33] Andò S., De Amicis F., Rago V., Carpino A., Maggiolini M., Panno M., Lanzino M. (2002). Breast cancer: From estrogen to androgen receptor. Mol. Cell Endocrinol..

[34] Lanzino M., De Amicis F., McPhaul M.J., Marsico S., Panno M.L., Ando S. (2005). Endogenous coactivator ARA70 interacts with estrogen receptor alpha (ERalpha) and modulates the functional ERalpha/androgen receptor interplay in MCF-7 cells. J. Biol. Chem..

[35] Lanzino M., Maris P., Sirianni R., Barone I., Casaburi I., Chimento A., Giordano C., Morelli C., Sisci D., Rizza P. (2013). DAX-1, as an androgen-target gene, inhibits aromatase expression: A novel mechanism blocking estrogen-dependent breast cancer cell proliferation. Cell Death Dis..

[36] Lanzino M., Sisci D., Morelli C., Garofalo C., Catalano S., Casaburi I., Capparelli C., Giordano C., Giordano F., Maggiolini M. (2010). Inhibition of cyclin D1 expression by androgen receptor in breast cancer cells--identification of a novel androgen response element. Nucleic Acids Res..

[37] Casaburi I., Cesario M.G., Donà A., Rizza P., Aquila S., Avena P., Lanzino M., Pellegrino M., Vivacqua A., Tucci P. (2016). Androgens downregulate miR-21 expression in breast cancer cells underlining the protective role of androgen receptor. Oncotarget.

[38] Rizza P., Barone I., Zito D., Giordano F., Lanzino M., De Amicis F., Mauro L., Sisci D., Catalano S., Wright K.D. (2014). Estrogen receptor beta as a novel target of androgen receptor action in breast cancer cell lines. Breast Cancer Res..

[39] Vasiliou S.K., Filippou P.S., Clotet-Freixas S., Soosaipillai A., Batruch I., Viktor Tsianos F., Konvalinka A., Diamandis E.P. (2022). Transcriptome profiling and proteomic validation reveals targets of the androgen receptor signaling in the BT-474 breast cancer cell line. Clin. Proteom..

[40] Hickey T.E., Dwyer A.R., Tilley W.D. (2021). Arming androgen receptors to oppose oncogenic estrogen receptor activity in breast cancer. Br. J. Cancer.

[41] Greeve M.A., Allan R.K., Harvey J.M., Bentel J.M. (2004). Inhibition of MCF-7 breast cancer cell proliferation by 5alpha-dihydrotestosterone; a role for p21(Cip1/Waf1). J. Mol. Endocrinol..

[42] Wang Y., Romigh T., He X., Tan M.-H., Orloff M.S., Silverman R.H., Heston W.D., Eng C. (2011). Differential regulation of PTEN expression by androgen receptor in prostate and breast cancers. Oncogene.

[43] Thompson C.B. (1995). Apoptosis in the pathogenesis and treatment of disease. Science.

[44] Fernald K., Kurokawa M. (2013). Evading apoptosis in cancer. Trends Cell Biol..

[45] Huang D.C., O’Reilly L.A., Strasser A., Cory S. (1997). The anti-apoptosis function of Bcl-2 can be genetically separated from its inhibitory effect on cell cycle entry. EMBO J..

[46] Yeretssian G., Correa R.G., Doiron K., Fitzgerald P., Dillon C.P., Green D.R., Reed J.C., Saleh M. (2011). Non-apoptotic role of BID in inflammation and innate immunity. Nature.

[47] Danial N.N., Gramm C.F., Scorrano L., Zhang C.Y., Krauss S., Ranger A.M., Datta S.R., Greenberg M.E., Licklider L.J., Lowell B.B. (2003). BAD and glucokinase reside in a mitochondrial complex that integrates glycolysis and apoptosis. Nature.

[48] Lowman X.H., McDonnell M.A., Kosloske A., Odumade O.A., Jenness C., Karim C.B., Jemmerson R., Kelekar A. (2010). The proapoptotic function of Noxa in human leukemia cells is regulated by the kinase Cdk5 and by glucose. Mol. Cell.

[49] Mann J., Githaka J.M., Buckland T.W., Yang N., Montpetit R., Patel N., Li L., Baksh S., Godbout R., Lemieux H. (2019). Non-canonical BAD activity regulates breast cancer cell and tumor growth via 14-3-3 binding and mitochondrial metabolism. Oncogene.

[50] Fernando R., Foster J.S., Bible A., Strom A., Pestell R.G., Rao M., Saxton A., Baek S.J., Yamaguchi K., Donnell R. (2007). Breast cancer cell proliferation is inhibited by BAD: Regulation of cyclin D1. J. Biol. Chem..

[51] Cekanova M., Fernando R.I., Siriwardhana N., Sukhthankar M., de la Parra C., Woraratphoka J., Malone C., Ström A., Baek S.J., Wade P.A. (2015). BCL-2 family protein, BAD is down-regulated in breast cancer and inhibits cell invasion. Exp. Cell Res..

[52] Zha J., Harada H., Yang E., Jockel J., Korsmeyer S.J. (1996). Serine phosphorylation of death agonist BAD in response to survival factor results in binding to 14-3-3 not BCL-X(L). Cell.

[53] Datta S.R., Ranger A.M., Lin M.Z., Sturgill J.F., Ma Y.C., Cowan C.W., Dikkes P., Korsmeyer S.J., Greenberg M.E. (2002). Survival factor-mediated BAD phosphorylation raises the mitochondrial threshold for apoptosis. Dev. Cell.

[54] Hu W., Fu J., Lu S.X., Liu L.L., Luo R.Z., Yun J.P., Zhang C.Z. (2015). Decrease of Bcl-xL/Bcl-2-associated death promoter in hepatocellular carcinoma indicates poor prognosis. Am. J. Cancer Res..

[55] Sinicrope F.A., Rego R.L., Foster N.R., Thibodeau S.N., Alberts S.R., Windschitl H.E., Sargent D.J. (2008). Proapoptotic BAD and Bid protein expression predict survival in stages II and III colon cancers. Clin. Cancer Res..

[56] Yancey D., Nelson K.C., Baiz D., Hassan S., Flores A., Pullikuth A., Karpova Y., Axanova L., Moore V., Sui G. (2013). BAD dephosphorylation and decreased expression of MCL-1 induce rapid apoptosis in prostate cancer cells. PLoS ONE.

[57] Craik A.C., Veldhoen R.A., Czernick M., Buckland T.W., Kyselytzia K., Ghosh S., Lai R., Damaraju S., Underhill D.A., Mackey J.R. (2010). The BH3-only protein BAD confers breast cancer taxane sensitivity through a nonapoptotic mechanism. Oncogene.

[58] Cannings E., Kirkegaard T., Tovey S.M., Dunne B., Cooke T.G., Bartlett J.M.S. (2007). BAD expression predicts outcome in patients treated with tamoxifen. Breast Cancer Res. Treat..

[59] Yu B., Sun X., Shen H.-Y., Gao F., Fan Y.-M., Sun Z.-J. (2010). Expression of the apoptosis-related genes BCL-2 and BAD in human breast carcinoma and their associated relationship with chemosensitivity. J. Exp. Clin. Cancer Res..

[60] Al-Bazz Y.O., Underwood J.C., Brown B.L., Dobson P.R. (2009). Prognostic significance of Akt, phospho-Akt and BAD expression in primary breast cancer. Eur. J. Cancer.

[61] Huang Y., Liu D., Chen B., Zeng J., Wang L., Zhang S., Mo X., Li W. (2012). Loss of BAD expression confers poor prognosis in non-small cell lung cancer. Med. Oncol..

[62] Troutaud D., Petit B., Bellanger C., Marin B., Gourin-Chaury M.-P., Petit D., Olivrie A., Feuillard J., Jauberteau M.-O., Bordessoule D. (2010). Prognostic significance of BAD and AIF apoptotic pathways in diffuse large B-cell lymphoma. Clin. Lymphoma Myeloma Leuk..

[63] Kitada S., Krajewska M., Zhang X., Scudiero D., Zapata J.M., Wang H.G., Shabaik A., Tudor G., Krajewski S., Myers T.G. (1998). Expression and location of pro-apoptotic Bcl-2 family protein BAD in normal human tissues and tumor cell lines. Am. J. Pathol..

[64] Gyorffy B., Lanczky A., Eklund A.C., Denkert C., Budczies J., Li Q., Szallasi Z. (2010). An online survival analysis tool to rapidly assess the effect of 22,277 genes on breast cancer prognosis using microarray data of 1,809 patients. Breast Cancer Res. Treat..

[65] Landes T., Martinou J.C. (2011). Mitochondrial outer membrane permeabilization during apoptosis: The role of mitochondrial fission. Biochim. Biophys. Acta.

[66] Papadopoulou N., Papakonstanti E.A., Kallergi G., Alevizopoulos K., Stournaras C. (2009). Membrane androgen receptor activation in prostate and breast tumor cells: Molecular signaling and clinical impact. IUBMB Life.

[67] Gu S., Papadopoulou N., Gehring E.-M., Nasir O., Dimas K., Bhavsar S.K., Föller M., Alevizopoulos K., Lang F., Stournaras C. (2009). Functional membrane androgen receptors in colon tumors trigger pro-apoptotic responses in vitro and reduce drastically tumor incidence in vivo. Mol. Cancer.

[68] Kampa M., Nifli A.-P., Charalampopoulos I., Alexaki V.-I., Theodoropoulos P.A., Stathopoulos E.N., Gravanis A., Castanas E. (2005). Opposing effects of estradiol- and testosterone-membrane binding sites on T47D breast cancer cell apoptosis. Exp. Cell Res..

[69] Liu G., Honisch S., Liu G., Schmidt S., Pantelakos S., Alkahtani S., Toulany M., Lang F., Stournaras C. (2015). Inhibition of SGK1 enhances mAR-induced apoptosis in MCF-7 breast cancer cells. Cancer Biol. Ther..

[70] Pelekanou V., Notas G., Sanidas E., Tsapis A., Castanas E., Kampa M. (2010). Testosterone membrane-initiated action in breast cancer cells: Interaction with the androgen signaling pathway and EPOR. Mol. Oncol..

[71] Hanahan D., Weinberg R.A. (2000). The hallmarks of cancer. Cell.

[72] Hanahan D., Weinberg R.A. (2011). Hallmarks of cancer: The next generation. Cell.

[73] Fouad Y.A., Aanei C. (2017). Revisiting the hallmarks of cancer. Am. J. Cancer Res..

[74] Lopez A., Reyna D.E., Gitego N., Kopp F., Zhou H., Miranda-Roman M.A., Nordstrøm L.U., Narayanagari S.-R., Chi P., Vilar E. (2022). Co-targeting of BAX and BCL-XL proteins broadly overcomes resistance to apoptosis in cancer. Nat. Commun..

[75] Frenzel A., Grespi F., Chmelewskij W., Villunger A. (2009). Bcl2 family proteins in carcinogenesis and the treatment of cancer. Apoptosis.

[76] Ranger A.M., Zha J., Harada H., Datta S.R., Danial N.N., Gilmore A.P., Kutok J.L., Le Beau M.M., Greenberg M.E., Korsmeyer S.J. (2003). BAD-deficient mice develop diffuse large B cell lymphoma. Proc. Natl. Acad. Sci. USA.

[77] Canevari R.A., Marchi F.A., Domingues M.A.C., de Andrade V.P., Caldeira J.R.F., Verjovski-Almeida S., Rogatto S.R., Reis E.M. (2016). Identification of novel biomarkers associated with poor patient outcomes in invasive breast carcinoma. Tumour Biol..

[78] Popgeorgiev N., Jabbour L., Gillet G. (2018). Subcellular Localization and Dynamics of the Bcl-2 Family of Proteins. Front. Cell Dev. Biol..

[79] Dumitru R., Gama V., Fagan B.M., Bower J.J., Swahari V., Pevny L.H., Deshmukh M. (2012). Human embryonic stem cells have constitutively active Bax at the Golgi and are primed to undergo rapid apoptosis. Mol. Cell.

[80] Hosoi K.I., Miyata N., Mukai S., Furuki S., Okumoto K., Cheng E.H., Fujiki Y. (2017). The VDAC2-BAK axis regulates peroxisomal membrane permeability. J. Cell Biol..

[81] Kamer I., Sarig R., Zaltsman Y., Niv H., Oberkovitz G., Regev L., Haimovich G., Lerenthal Y., Marcellus R.C., Gross A. (2005). Proapoptotic BID is an ATM effector in the DNA-damage response. Cell.

[82] Bonneau B., Prudent J., Popgeorgiev N., Gillet G. (2013). Non-apoptotic roles of Bcl-2 family: The calcium connection. Biochim. Biophys. Acta.

[83] Choi S., Chen Z., Tang L.H., Fang Y., Shin S.J., Panarelli N.C., Chen Y.T., Li Y., Jiang X., Du Y.N. (2016). Bcl-xL promotes metastasis independent of its anti-apoptotic activity. Nat. Commun..

[84] Schwartz N., Verma A., Bivens C.B., Schwartz Z., Boyan B.D. (2016). Rapid steroid hormone actions via membrane receptors. Biochim. Biophys. Acta.

[85] Kousteni S., Bellido T., Plotkin L.I., O’Brien C.A., Bodenner D.L., Han L., Han K., DiGregorio G.B., Katzenellenbogen J.A., Katzenellenbogen B.S. (2001). Nongenotropic, sex-nonspecific signaling through the estrogen or androgen receptors: Dissociation from transcriptional activity. Cell.

[86] Almeida M., Han L., O’brien C.A., Kousteni S., Manolagas S.C. (2006). Classical genotropic versus kinase-initiated regulation of gene transcription by the estrogen receptor alpha. Endocrinology.

[87] Grazzini E., Guillon G., Mouillac B., Zingg H.H. (1998). Inhibition of oxytocin receptor function by direct binding of progesterone. Nature.

[88] Vertino A.M., Bula C.M., Chen J.R., Almeida M., Han L., Bellido T., Kousteni S., Norman A.W., Manolagas S.C. (2005). Nongenotropic, anti-apoptotic signaling of 1alpha,25(OH)2-vitamin D3 and analogs through the ligand binding domain of the vitamin D receptor in osteoblasts and osteocytes. Mediation by Src, phosphatidylinositol 3-, and JNK kinases. J. Biol. Chem..

[89] Hammes S.R., Levin E.R. (2007). Extranuclear steroid receptors: Nature and actions. Endocr. Rev..

[90] Pedram A., Razandi M., Sainson R.C., Kim J.K., Hughes C.C., Levin E.R. (2007). A conserved mechanism for steroid receptor translocation to the plasma membrane. J. Biol. Chem..

[91] Danial N.N., Walensky L.D., Zhang C.-Y., Choi C.S., Fisher J.K., Molina A.J., Datta S.R., Pitter K.L., Bird G.H., Wikstrom J.D. (2008). Dual role of proapoptotic BAD in insulin secretion and beta cell survival. Nat. Med..

[92] Gimenez-Cassina A., Garcia-Haro L., Choi C.S., Osundiji M.A., Lane E.A., Huang H., Yildirim M.A., Szlyk B., Fisher J.K., Polak K. (2014). Regulation of hepatic energy metabolism and gluconeogenesis by BAD. Cell Metab..

[93] Cheng W.-C., Leach K.M., Hardwick J.M. (2008). Mitochondrial death pathways in yeast and mammalian cells. Biochim. Biophys. Acta.

[94] Lee Y.G., Nam Y., Shin K.J., Yoon S., Park W.S., Joung J.Y., Seo J.K., Jang J., Lee S., Nam D. (2020). Androgen-induced expression of DRP1 regulates mitochondrial metabolic reprogramming in prostate cancer. Cancer Lett..

[95] Lanzino M., Garofalo C., Morelli C., Le Pera M., Casaburi I., McPhaul M.J., Surmacz E., Andò S., Sisci D. (2009). Insulin receptor substrate 1 modulates the transcriptional activity and the stability of androgen receptor in breast cancer cells. Breast Cancer Res. Treat..

