# Supplementary material for: Androgens Modulate Bcl-2 Agonist of Cell Death (BAD) Expression and Function in Breast Cancer Cells
Source: Int J Mol Sci. 2023 Aug 30;24(17):13464. doi: 10.3390/ijms241713464 (PMC10487823; doi:10.3390/ijms241713464)
Supplement: Supplementary file 1 [file ijms-24-13464-s001.zip › ijms-2544666-supplementary.pdf]

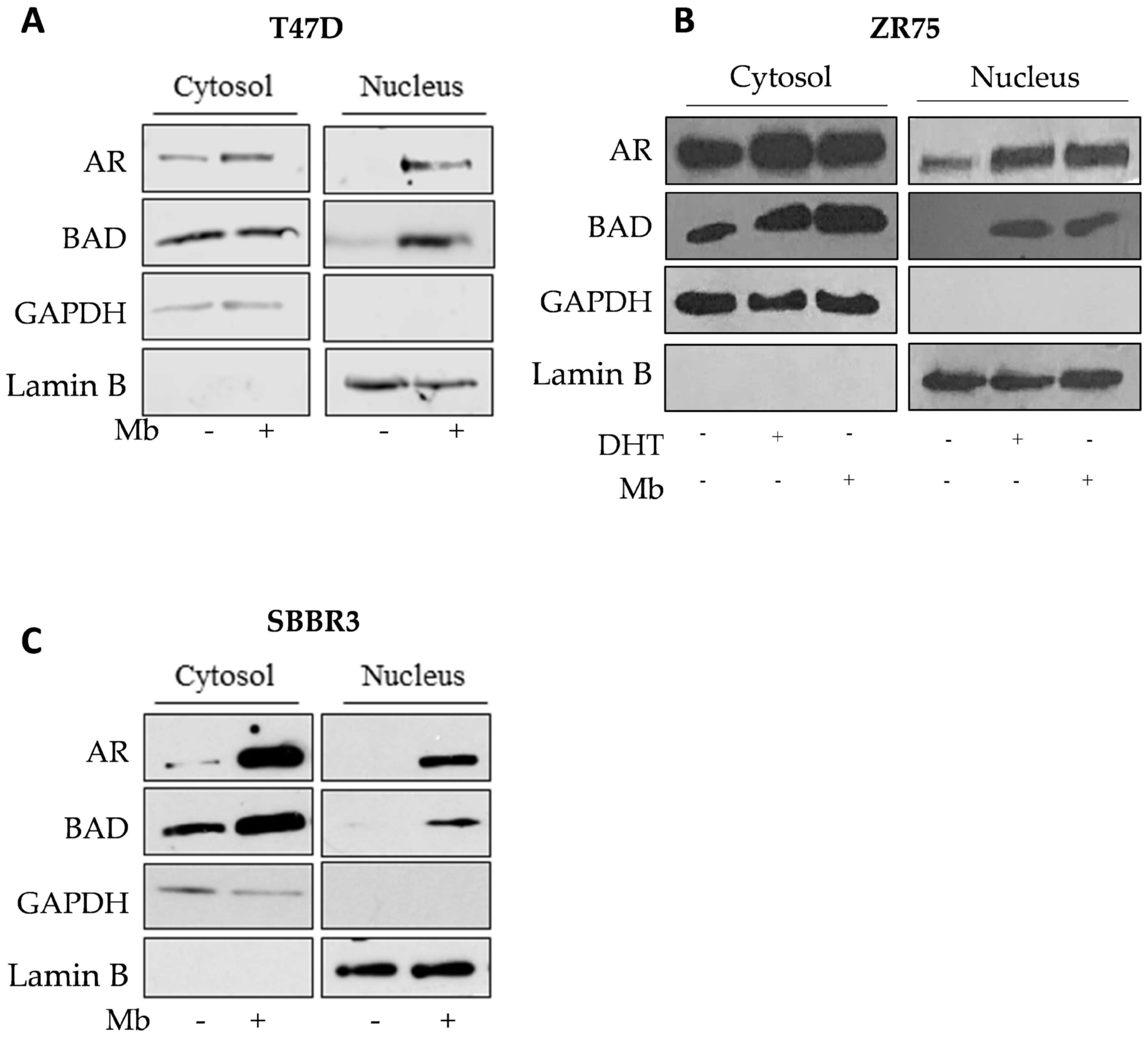

Figure S1. Androgens induce BAD nuclear localization. Western Blotting analysis of cytoplasmic and nuclear protein extracts from T47D **(A)**, ZR75 (**B**) and SKBR3 **(C)** breast cancer cells treated for 24h with vehicle (-) or 10 nM DHT as indicated. LAMIN B and GAPDH were used as loading control.

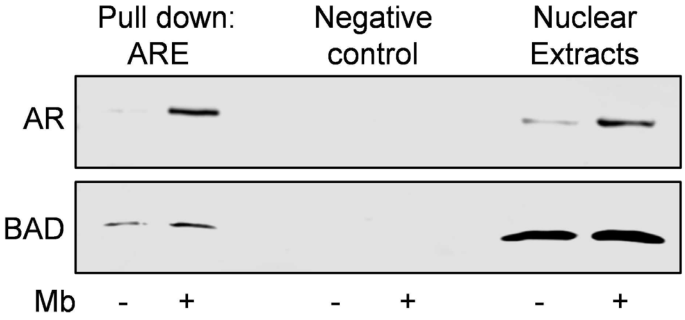

Figure S2. Androgens induce AR and BAD recruitment to CCND1-ARE site. Nuclear extract from T47D cells treated with 10 nM Mb for 2h or vehicle (-) were incubated with a biotinylated oligonucleotide containing the CCND1-ARE site, and subjected to DAPA. Specifically-bound proteins were subjected to western blotting analysis using anti-AR and anti-BAD antibodies. Unbound fraction was loaded as negative control; nuclear extracts were loaded as positive control.
